# eGIFT: Mining Gene Information from the Literature

**DOI:** 10.1186/1471-2105-11-418

**Published:** 2010-08-09

**Authors:** Catalina O Tudor, Carl J Schmidt, K Vijay-Shanker

**Affiliations:** 1 Department of Computer and Information Sciences, University of Delaware, Newark, Delaware, USA; 2 Department of Animal and Food Sciences, University of Delaware, Newark, Delaware, USA

## Abstract

**Background:**

With the biomedical literature continually expanding, searching PubMed for information about specific genes becomes increasingly difficult. Not only can thousands of results be returned, but gene name ambiguity leads to many irrelevant hits. As a result, it is difficult for life scientists and gene curators to rapidly get an overall picture about a specific gene from documents that mention its names and synonyms.

**Results:**

In this paper, we present eGIFT (http://biotm.cis.udel.edu/eGIFT), a web-based tool that associates informative terms, called *i*Terms, and sentences containing them, with genes. To associate *i*Terms with a gene, eGIFT ranks *i*Terms about the gene, based on a score which compares the frequency of occurrence of a term in the gene's literature to its frequency of occurrence in documents about genes in general. To retrieve a gene's documents (Medline abstracts), eGIFT considers all gene names, aliases, and synonyms. Since many of the gene names can be ambiguous, eGIFT applies a disambiguation step to remove matches that do not correspond to this gene. Another additional filtering process is applied to retain those abstracts that focus on the gene rather than mention it in passing. eGIFT's information for a gene is pre-computed and users of eGIFT can search for genes by using a name or an EntrezGene identifier. *i*Terms are grouped into different categories to facilitate a quick inspection. eGIFT also links an *i*Term to sentences mentioning the term to allow users to see the relation between the *i*Term and the gene. We evaluated the precision and recall of eGIFT's *i*Terms for 40 genes; between 88% and 94% of the *i*Terms were marked as salient by our evaluators, and 94% of the UniProtKB keywords for these genes were also identified by eGIFT as *i*Terms.

**Conclusions:**

Our evaluations suggest that *i*Terms capture highly-relevant aspects of genes. Furthermore, by showing sentences containing these terms, eGIFT can provide a quick description of a specific gene. eGIFT helps not only life scientists survey results of high-throughput experiments, but also annotators to find articles describing gene aspects and functions.

## Background

Biomedical literature is expanding quickly. Main literature sources, such as PubMed [1] and BioMed Central [2], contain millions of articles and continue to grow daily. As a result, life scientists spend considerable time searching the literature for gene or protein-specific information. In this paper, we present **eGIFT **(**E**xtracting **G**ene **I**nformation **F**rom **T**ext), a tool which automatically identifies salient information about a gene and its products.

The main motivation for eGIFT is to minimize time required for researchers to learn about an unfamiliar gene, or to find gene-related information from the biomedical literature. In particular, the objective is (1) to provide life scientists with a rapid means to grasp important properties or functions of genes, and (2) to assist in understanding specific aspects of genes, by directing users to pertinent text passages. eGIFT extracts terms for a gene by comparing their frequencies in a set of a gene's documents with their frequencies in a background set. These terms, which we call *i*Terms (informative terms) provide a biologist with a synoptic understanding of a gene. *i*Terms are directly linked to sentences in the gene's abstracts that help a biologist better place them in a biological context.

For example, consider how eGIFT can assist a researcher survey the results of a high-throughput expression study. The researcher notices an unfamiliar gene, *Groucho*, that is differentially expressed, and searches for abstracts that mention word *Groucho*. The researcher might use the synonym *GRO *to expand the search, thus yielding thousands of results. In this scenario, many results will be irrelevant, since *GRO *is ambiguous and could represent other entities such as: growth-related oncogene, or General Register Office. Unless the researcher reads a large portion of the abstracts, she will not get an overall understanding of the gene.

In contrast, when searching eGIFT for *Groucho*, the researcher is provided with a list of terms that eGIFT marks as highly relevant to the gene (see Additional File 1 for a screenshot of eGIFT's *i*Terms for *Groucho*). For example, eGIFT's top *i*Terms for *Groucho *include *transcriptional corepressor, segmentation, neurogenesis, WD40 *and *wprw*. These *i*Terms allow the researcher to infer that *Groucho *is likely a *transcriptional corepressor*, and that it might be involved in the processes of *segmentation *and *neurogenesis*. For each term in the list, one click away are sentences that confirm the inference. Thus, clicking on the remaining terms, *WD40* and *wprw*, the researcher learns that *Groucho* contains the *WD40* domain and interacts with proteins that contain the *wrpw *motif. By showing *i*Terms and sentences containing them, we summarize the most important information from a gene's literature.

The rest of the paper is organized as follows: We start by describing related work. Then, we follow with details about eGIFT's implementation. We continue with the evaluation of eGIFT, followed by a discussion of eGIFT's results and the quality of its *i*Terms. Finally, we draw conclusions and present future work.

## Related Work

One of the early works on mining important terms for gene/protein families was proposed by Andrade and Valencia [3]. They automatically mine such terms from the biomedical literature by computing scores for each word in a given protein family. The scores are based on the frequency of the word in the family, the average frequency of the word, and the deviation of word distribution over all families (Z-score). Liu et al. [4] extended this method to statistically mine functional terms associated with genes, and conducted a case study on *OPN*. A more recent system, HT-SAS [5], collects abstracts annotated in UniProt for a gene, and determines common words co-occurring with the gene in these documents.

EBIMed [6], FACTA [7], PolySearch [8], and Lit-Miner [9] also identify important terms co-occurring with genes in the biomedical literature. These systems, in contrast to the ones mentioned earlier, only consider terms limited to certain controlled vocabularies. They use different scoring methods (all based on frequency of co-occurrence with the gene) to rank the terms. These four methods also differ in the types of terms and controlled vocabularies they use.

Systems such as e-LiSe [10] and MedEvi [11] do not restrict themselves to finding important terms correlated with genes. Instead, their search can be of any category and these systems use the Z-score to identify important terms for the given query. Similarly, Anne O'Tate [12] retrieves articles from PubMed, based on a boolean query provided by the user, and then displays important words, topics (based on MeSH terms), clusters (by topic), authors, affiliations, journals, and years. Somewhat related are also the works of Iratxeta et al. [13] and Shatkay and Wilbur [14], who determine important words in a set of documents.

Some systems attempt to identify relations between genes or clusters of genes. In general, for two genes to be related, they need to co-occur significantly in the biomedical literature, or they need to co-occur with similar concepts. Anni 2.0 [15] focuses on creating profiles (based on controlled vocabulary terms) for genes and relates genes based on these profiles. Similarly, Tsoi et al. [16] create ontology fingerprints for genes based on Gene Ontology terms that are co-mentioned frequently in their literature. These fingerprints are then used to identify similarities among genes. GeneNarrator [17] takes as input a list of genes, collects abstracts from PubMed and clusters these abstracts. The genes themselves are then grouped based on the distribution of occurrences in the different document clusters.

## Implementation

The four major parts of eGIFT are described below. The steps are fully automatic, and no manual intervention is needed.

### 1. Retrieving documents for a gene

**Figure 1:**
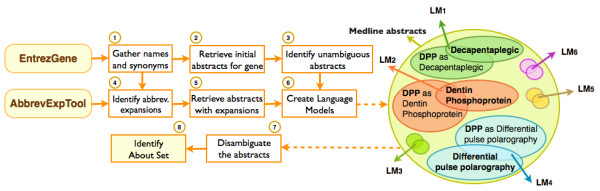
**eGRAB module for retrieving Medline abstracts about a gene**. Gene names and aliases are gathered from EntrezGene to retrieve Medline abstracts. eGRAB filters out abstracts that mention an ambiguous gene name in some other context. This is done by creating bigram language models for each sense of an ambiguous name and picking the language model that best fits an abstract. **DPP **is used here to illustrate the ambiguity of gene names (DPP stands for two different genes - Decapentaplegic and Dentin Phosphoprotein - as well as a technique - Differential polarography, among multiple other senses).

The following steps are used in obtaining an initial set of abstracts for a gene (see Figure 1):

1. A gene's identifier or official names (as used by EntrezGene) are input to the system (these are not restricted to any specific species).

2. Synonyms of the gene are gathered from all species (variations in names are considered to account for inclusion/exclusion of hyphenation and spacing [18], and synonyms are restricted to the ones found in entries for Homo sapiens, Mus musculus, Rat rattus, or at least two other species).

3. All names, synonyms, and variations of these are used as an expanded query to retrieve Medline abstracts (for the retrieval, we use an in-house implementation of the Lucene search engine [19]).

We choose abstracts because they are not only more readily available than full text, but are also condensed descriptions focusing on what is central to the study, combining the background, results and conclusions succinctly. Our reasoning is consistent with the observations in Shah et al. [20], who show that the abstract section of articles contains the best proportion of keywords, while the other sections are a better source of biologically relevant data.

#### Disambiguation of gene names

While the use of synonyms increases the recall, it can lead to a drop in precision if care is not taken to eliminate irrelevant retrievals. For instance, *GRO *is a short name/symbol (as used in Uniprot and Entrez Gene respectively) for *Groucho*, but within Medline, it has been used with multiple senses, including "growth related oncogene", "gasoline range organics" and "global repair operator". We use surrounding words to disambiguate between multiple senses of a word, which is a standard NLP method [21,22] that has been used for disambiguation of biomedical abbreviations (e.g. [23,24]). Since it is not possible to have manually annotated data for every possible gene name, we create the training set automatically by considering only those cases that can be assigned to a sense with high confidence. These high confidence cases are identified by insisting that an abstract mentions two or more names of a particular gene, such as "gro" and "Groucho" for example. On the other hand, abstracts which mention "gro" and "gasoline range operators" are assigned with high confidence to this other sense of "gro".

The basic steps for determining the abstracts that mention a given gene are:

1. Identify non-ambiguous abstracts (due to the appearance of multiple different names of the gene, as explained above).

2. Identify all possible expansions for names of the gene and retrieve Medline abstracts for each expansion.

3. Create language models for the set of abstracts identified in steps 1 and 2.

4. For every abstract initially retrieved for the gene names, choose the language model that best represents the abstract.

Typically, abbreviations are highly ambiguous. In step 2, we use the algorithm developed by Schwartz et al. [25] to detect all abbreviation-expansion pairs from text. For each sense (expansion) of an abbreviated name, we gather all the abstracts mentioning the expansion and its lexical variations, and identify those abstracts where the abbreviation-expansion pairs are detected by the tool with high confidence. Thus, given a set of abstracts for each expansion, a bigram language model is formed for each sense in step 3. When a new abstract is encountered that mentions only a short name (with no expansion defined), all the language models are applied, and the most likely sense is chosen (step 4).

The set of all abstracts associated with a gene, using this method, will be called the **Full Set **for the gene. Since the document retrieval and disambiguation steps can be used in many other applications besides eGIFT, they are now implemented as a stand-alone module, called **eGRAB **(**E**xtractor of **G**ene-**R**elevant **AB**stracts), illustrated in Figure 1.

To assess how well eGRAB completes its task, we evaluated its retrieval on 40 genes (see Additional File 2 for the list of genes). eGRAB's ability to disambiguate correctly can be calculated in terms of accuracy, precision, and recall. First, for each gene, we randomly selected 20 abstracts returned by PubMed on the gene and its synonyms, and manually marked whether they represented the gene and its synonyms. Here, we report an accuracy of 86.38%. For precision, we selected a different random set of 20 abstracts that eGRAB marked as relevant for each gene. We manually marked true positives and true negatives in these sets and obtained a precision of 86.63%. For recall, we looked at how many abstracts were marked as relevant when compared to the EntrezGene PubMed links (gene2pubmed), thus obtaining a recall of 91.85%.

#### Filtering noisy documents

We had noticed that usually the gene that is the focus of an abstract is either mentioned frequently or appears in textual places such as the title, the first, or the last sentence. With the assumption that genes that are mentioned in passing rarely have such occurrences, we pick those abstracts from the Full Set that mention the gene of interest at least three times and/or mention the gene in the title, the first, or the last sentence. This subset of abstracts will be called the **About Set **for the gene.

We evaluated how well our heuristics aid in identifying highly-relevant abstracts for a gene. Given an abstract and a gene, the task was to determine whether the gene plays a significant role in the study reported in the abstract. For example, gene *BMP2 *is the focus of abstract with title: "Bmp2 is essential for postnatal osteogenesis but not for recruitment of osteogenic stem cell" (PMID 19398043), while gene *Groucho *is only mentioned in passing in abstract "Formation and patterning of the forebrain and olfactory system by zinc-finger genes Fezf1 and Fezf2" (PMID 19222525). For this evaluation, we randomly gathered a set of abstracts from the literature on the same 40 genes as used in the evaluation of eGRAB, and asked 5 judges (other than the authors of this paper) to make this determination. Disregarding the abstracts where the judges indicated that they were unsure (2 abstracts), we report an agreement of 88.02% between the judges evaluation and our rules, for 167 abstracts.

### 2. Identifying *i*Terms

eGIFT assigns scores to **unigrams **(single-word terms), **bigrams **(two-word terms) which do not contain stopwords, as well as a set of biomedical terms that we extracted from different knowledge bases, including EntrezGene, Gene Ontology, NCBI Taxonomy, UMLS, and MeSH that matched in text. Instead of considering words as the unit for terms, we consider *lexemes*. Lexemes are formed by grouping words with the same stem since typically they express the same concept. For example, the frequency of the unigram lexeme "repression" will contain the frequency of occurrence of words: *repression, repress, repressed, repressing, represses, repressor*, and *repressors*. To identify the lexemes, we have built our own morphological processor that accounts for different inflections using the methodology described in Miller et al. [26]. The lexeme representation is used for both unigrams and bigrams.

**Figure 2:**
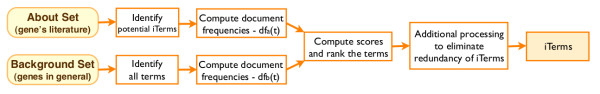
**Identifying ***i***Terms**. *i*Terms are obtained by ranking important terms based on a score which combines their Background Set and About Set document frequencies. We also attempt to eliminate lexical redundancies among *i*Terms before displaying them.

Each term is assigned a score that contrasts the frequency of occurrence of the term in the given gene's About Set with the frequency of the term in a background set (see Figure 2). For the latter, we downloaded Medline abstracts whose titles contain the words *gene(s) *and *protein(s)*. We call this set the **Background Set**, which currently has a total of 639,211 non-empty abstracts.

To identify *i*Terms, for each term *t*, a score *s*(*t*) is assigned as follows:

where *df_a_*(*t*) and *df_b_*(*t*) are the number of abstracts containing term *t *in the About Set for the gene and the Background Set, respectively, and *N_a _*and *N_b _*are the total number of abstracts in these two sets. Note that we have not used the frequency of the term in the set of abstracts, but rather the document frequency (df), as we are more interested in relevance of a term to a gene's set of abstracts, rather than relevance to a single document.

Since the number of abstracts for a gene can vary significantly and is smaller than that of the back-ground set, the document frequencies are normalized. In some cases, this normalized frequency can be small (especially for the background set where there is a large denominator). But small changes have large impact when taking their ratio, so we consider the difference between the normalized document frequencies .

The difference works well when the terms are equally distributed in the background. However, there are cases when the background frequency of a term is very large, and hence suggesting the term is not highly discriminatory among genes. To overcome this, we use , which is used in document retrieval systems to penalize frequent words. For example, terms *segmentation *and *these *are both mentioned in gene *Groucho*'s abstracts, equally more than in the background set (i.e., they both have the same difference of 0.13). The second part of the equation dampens the difference. As a result, the scores for the terms *segmentation *and *these *for *Groucho *diverges (0.874 and 0.098 respectively), making *segmentation *a highly ranked term and dropping *these *significantly.

Because the background set is so large, certain infrequent bigrams will have a very high score due to the second part of the equation. Hence, we consider only those bigrams that meet a minimum threshold of 10 abstracts in the background set (i.e. *df_b _*for this bigram is at least 10).

Next, we apply rules to address **redundancy**. Some words frequently appear together (e.g. "lymphoblastic leukemia" for *LMO2*). Rather than list *lymphoblastic *and *lymphoblastic leukemia *separately (if their ranks are high), we remove the unigram in favor of the bigram, which we take to be more informative. This rule is applicable only when the bigram occurs 75% of the times the unigram occurs alone. For instance, it is not applied for *Groucho*'s *i*Term *repressor*. Although about 50% of these occurrences are within *transcriptional repressor*, there are other useful bigrams it appears with (e.g., *repressor domain*). Using this rule, we eliminated on average 5% of redundant *i*Terms from eGIFT's results.

### 3. Categorization of *i*Terms

Different researchers may have different interests. For example, a clinical researcher might be interested in learning about diseases in which a gene plays an important role. Conversely, a GO annotator might be interested in functional terms and processes to deduce a possible annotation for the gene. To facilitate a quick inspection of different aspects of interest, *i*Terms are segregated into different categories and presented in descending order within each category. Much of our categorization is based on the use of different ontologies and controlled vocabularies (UMLS, MeSH, Gene Ontology, UniProt keywords, and NCBI's controlled vocabularies), as well as on word endings and cue words for different categories. The ontologies and controlled vocabularies used for each particular category, as well as the cue words and word endings, are listed on eGIFT's website.

We divide eGIFT's categories into *primary *and *secondary *categories. The primary categories are *Functions and Processes; GO Related Terms; Domains and Motifs; Pathways and Signaling*; and *Diseases*. The secondary categories are: *Gene (Family) Names; Drugs and Chemical Compounds*; *Species Names; Anatomical Parts; Cells, Cell Types, and Cell Lines; Techniques and Treatments*. The terms that we could not classify are grouped together into an *Unclassified *category.

*i*Terms in primary categories usually provide a user with a high-level, overall understanding of the gene. On the other hand, *i*Terms from secondary categories typically provide information about certain details, such as what cell lines, or tissues, or experimental methods were used often in context of studies involving this gene. However, they could still be useful as gateways to relevant literature, through the sentences that contain them, to specific information needs, and hence are not discarded. We have previously shown how eGIFT might not be the right way to find other important genes to be related to the given gene [27] (and the reason applied equally to the remaining category of drug/compound). Using a frequency-based approach, eGIFT extracts as *i*Terms genes which tend to be similar to the given gene. For identifying interacting partners, however, other methods are better suited (such as information extraction methods). In [27], we have also shown how varying the notion of what the document query set is for a gene, different types of *i*Terms can be better extracted. For example, restricting the query set to only those sentences in which the given gene is mentioned, we were able to identify in a greater number genes that are important to the target gene.

### 4. Presentation of results

**Figure 3:**
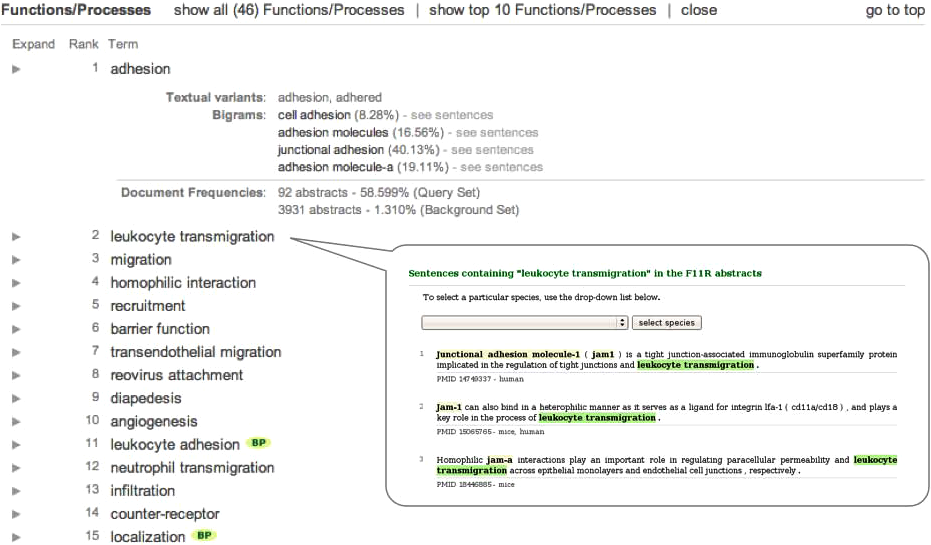
**Partial ***i***Terms of type functions/processes for gene F11R (JAM-1 or JAM-A)**. Extra information can be obtained for an *i*Term, by clicking the arrow to the left of it (see *adhesion *above). This information includes textual variants, most co-occurring adjacent terms (with which it forms bigrams), and frequencies in the Background and Query/About Sets. Ranked sentences can be retrieved by clicking on the term (see *leukocyte transmigration *above).

eGIFT's database contains precomputed *i*Terms and their categorization. A user wishing to learn about a gene will start by specifying the gene in eGIFT's search page. The user can query using any of the gene's names, aliases, or synonyms (that can be found in EntrezGene), part of a name, or an Entrez-Gene ID. All genes in eGIFT's database that match the query are displayed with their full names. The user has to choose the desired gene from the list, and then *i*Terms for that gene are presented to the user. Figure 3 shows part of the display for gene *F11R*, focusing on *i*Terms of type function/process.

By clicking the arrow at the left, eGIFT shows more information about the *i*Term, including textual variants in the lexeme, frequently occurring bigrams in case the *i*Term is a unigram (like *adhesion *in Figure 3, and its bigrams - *cell adhesion, junctional adhesion*, and *adhesion molecule-a*), and document frequencies. Sometimes a unigram may be too general and it might contain a few very useful bigrams, some of which may be *i*Terms themselves. These bigrams may sometimes be ranked lower because authors use different order of words to express the same idea. For example, *transcriptional activation *and *activation of transcription *are the same concept. While eGIFT extends to lexemes, these two are not considered equivalent. Thus, eGIFT assigns a lower frequency to the concept of *transcriptional activation*.

By clicking on the *i*Term itself, or any bigram shown in the expanded box for a unigram *i*Term, users can see ranked sentences (see pop-up window in Figure 3) with the *i*Term and target gene highlighted in each sentence. The ranking of sentences is rudimentary: it essentially ranks sentences higher if the gene occurs in the initial (subject) position of a sentence and if the *i*Term is in close proximity to the gene mention. These sentences facilitate a user's understanding of *i*Terms. For example, sentences will inform a user that *Groucho*'s *i*Term *wd40 *is a domain within *Groucho *and that *i*Term *wrpw *is a motif within other genes that interact with *Groucho*.

Any frequency-based approach will yield misleading results for low frequencies. This is why the set of documents for a gene is obtained without restricting the search for a specific species, since the literature for many gene-species pairs will be sparse. The core properties of a gene are likely to be common to many species and these will be captured as top-ranking terms in a species-independent approach. However, since some of the genes properties may be species specific, eGIFT allows a user to identify the *i*Terms and associated sentences that appear in the literature for the species of their interest. When a species is selected, the *i*Terms appearing in that species literature are highlighted and the linked species-specific sentences can be accessed by clicking on the *i*Term. Presently, eGIFT points only to the following species: Homo sapiens, Gallus gallus, Bos taurus, Mus musculus, and Sus scrofa.

Currently, species analysis is limited. eGIFT only looks for specific species names mentioned in either the title, abstract or MESH terms. The inference is made that the abstract describes attributes of the target gene in the context of the mentioned species. This inference, however, does not imply a linkage between a gene mention and a species in all cases. No additional effort is made to resolve species-gene relationships, and the biologist can determine if eGIFT made the correct inference. The association of species is an important topic of ongoing research [28,29]. Any efficient and accurate method that is developed can be integrated into eGIFT later.

#### Computational issues

Depending on the number of senses for each synonym of a gene, the disambiguation process can be time-consuming. Thus, like all search engines, we have taken the approach of pre-computing the *i*Terms and their categorization for the genes so that the results become immediately available to users.

## Results

Two evaluation studies were conducted:

(1) We evaluated how salient the *i*Terms are for a gene. Such an evaluation could be considered as measuring the *precision *of eGIFT.

(2) We also evaluated how many keywords, from existing annotations, eGIFT can extract. This second evaluation corresponds to measuring the *recall *of eGIFT for the task of extracting keywords.

### Experiment 1: Salience of *i*Terms (Precision)

A major objective of eGIFT is to assist a researcher in recognizing the biomedical and molecular properties associated with a gene. Hence, our first evaluation study was designed to see how many of the top ranked *i*Terms are indeed salient to the target genes. Given this objective of eGIFT and prior discussion of the difference between primary and secondary categories, we decided to only consider *i*Terms from primary and unclassified categories for the evaluation.

This evaluation was conducted for 40 genes (the genes and their results are provided as additional material - see Additional Files 2 and 3). For each gene, the top 20 ranked *i*Terms in these categories were considered. Since a researcher familiar with a gene may still not know all its properties, we allowed access to sentences containing the *i*Terms. The evaluators were encouraged (although not required) to look at the *i*Terms' sentences before rating them. Moreover, because the associated bigrams sometimes provide a better context to understand the salience of a unigram *i*Term, we provided the evaluators with these bigrams, as well as sentences containing them.

We selected 5 evaluators from different institutions (none of whom is involved in the design and development of eGIFT) to participate in this evaluation. The subjects have several years of biology research experience and/or several years of professional annotation experience. Since this process can be very time consuming, we asked them to provide a list of genes they were familiar with, for a total of 40 genes.

The evaluators were asked to pick one of three choices: salient, not salient, or unsure. We encouraged them to choose between the first two options, and limit the use of the unsure choice. Our annotators marked 87.6% of the evaluated *i*Terms as salient. Thus, on average, 17.5 of the 20 *i*Terms for a gene were found to be salient. In addition, 6.2% were marked with the unsure choice (which can still be relevant to the gene, but perhaps not as important). Only the remaining 6.2% were marked as not salient.

### Experiment 2: Recall of keywords

Even though the notion of *i*Terms is broader than that of terms found in controlled vocabularies and ontologies, we still wanted to determine how well eGIFT can assist with the GO term annotation task. Hence, for the same 40 genes as in Experiment 1, we picked terms already annotated for these genes, and verified how many of them were extracted by eGIFT. Rather than using GO terms for such a recall evaluation, we chose UniprotKB keywords. The reason lies in the observation that GO terms rarely occur in text [30]. Thus, recall of GO terms is not appropriate since eGIFT extracts terms that appear in text. Additionally, *i*Terms are one or two words long (more like UniProtKB keywords), whereas a majority of GO terms annotated for genes in knowledge bases are long. We also note that there is a mapping in UniProtKB from these keywords to GO terms.

For each of the 40 genes, we picked UniProtKB molecular function and biological process keywords from human entries. We chose human entries because human is the model organism with priority in UniProtKB manual curation pipeline and curators propagate annotations at least among mammalian proteins. Eight of the 40 genes did not have these types of keywords in their UniProtKB entries and, thus, were replaced by randomly selected genes. In total, we gathered 110 keywords for the 40 genes, or on average, 2.75 keywords per gene.

While keywords are often found in text, we also noticed that some matches were close but not exact. For example, keyword "transferase" was matched to *i*Term "kinase", because a kinase is a particular type of transferase. Hence, we allowed an *i*Term to be positively matched with a keyword provided one of the three conditions were met: (1) the *i*Term is the same as the keyword; (2) the *i*Term is a synonym of the keyword; (3) the *i*Term represents a specialization of the keyword (e.g. kinase-transferase). We did not allow for a match between highly-related terms not covered by the above rules, because we felt that it would introduce too much subjectivity. Since the guidelines were very specific, one of the co-authors, with extensive knowledge in biology, did this matching manually. We confirmed each match with two other professional annotators. In every case, there was a unanimous agreement.

We report a recall of 93.64%, which means that 103 of the 110 keywords were matched with corresponding *i*Terms. Of these, a little more than 70% were exact matches, and the remaining matched *i*Terms were synonyms or more specific terms.

## Discussion

Based on these evaluations, we believe that eGIFT performs well for its intended usage. These evaluations also provide us with some insights about eGIFT. We will discuss errors identified in the evaluations, as well as discuss differences between *i*Terms selection and gene annotations in knowledge bases.

### Error Analysis

Because *i*Terms are normally frequently occurring terms in a gene's document set, this approach can miss some important properties of the gene that are mentioned infrequently in the gene's literature. For example, *exonuclease *is a UniProt keyword for *TDP1*, but this term was not picked as an *i*Term for this gene, as it appears only once in the gene's literature. For the same reason, eGIFT and other frequency-based approaches to selection of informative terms might not mine recently discovered aspects of genes until they get mentioned sufficiently to be picked as *i*Terms. We plan on exploring taking into account the recency of publication in our future work to address this concern.

Because eGIFT considers relative frequency of terms (i.e., normalized by the document set size), its selection is susceptible to genes with a smaller query set. A term with few occurrences in a gene's document set can still have a relatively large normalized frequency. Typically, we have found that this limitation is not noticeable when the gene has at least 50 documents. Out of the 40 genes we used in our evaluation, two genes had less than 30 abstracts and two other genes had less than 50 abstracts, and their precision of *i*Terms was below average.

We also observed some problematic *i*Terms that appear frequently in the gene's document set. For example, the *i*Term *coagonist *appears frequently enough in *Serine racemase *(*SRR*)'s document set to be highly ranked. But this *i*Term was annotated as not relevant by our evaluators. Even with such cases, we find that sentences associated with the *i*Terms can still provide useful information. For example, "Serine racemase (sr) generates d-serine, a coagonist with glutamate at nmda receptors" (PMID 17293453) is one of the sentences associated with this term by eGIFT. While *SRR *is not a coagonist itself, this sentence reveals its relation to the *i*Term: *SRR *enzymatically synthesizes a coagonist.

While our evaluation of eGIFT and its components showed good precision and recall, we also noticed during the evaluation that some of the mistakes can be attributed to errors of the eGRAB module. For example, in the case of gene *CIB1*, eGRAB did not filter out all irrelevant abstracts containing a synonymous name, *Kip1*, which is also a synonym for *p27*, a cell cycle inhibitor. Although *Kip1 *was listed as a synonym for gene *CIB1*, no expansion of *Kip1 *could be found in the names of this gene. In fact, no expansion of any type could be determined for *Kip1 *in the literature, and so we assigned all abstracts mentioning *Kip1 *to *CIB1*'s sense by default. As a consequence, terms such as *cell cycle *and *localization *were erroneously identified as *i*Terms for *CIB1*.

We also evaluated the accuracy of heuristics used in the selection of the About Set. While the results are reasonably good, the analysis of errors suggests that some refinements to the rules may be needed. An abstract is currently included in the About set of a gene if the gene's name appears in the first or last sentence of the abstract. However, there were a few examples where the occurrence in the first or the last sentence was the only occurrence of the gene and the evaluators had found them to be not "about" the gene (e.g. PMID 19348653 for gene *HDAC2 *and PMID 16716254 for gene *FOXP2*). Also the current set of rules do not take into account the family name (e.g., *BMP*) of the gene (*BMP2*) when counting the number of occurrences throughout the abstract. Taking the family name into account might improve the recall of these rules.

While the evaluation did not address the issue of redundancy, we feel there is scope for additional work to address this issue. Although eGIFT collapses terms if they belong to the same lexeme family, it does not consider semantic relatedness. Hence, it is possible that two or more terms describing the same concept may be mentioned often enough in the literature that they are selected as *i*Terms, thus leading to redundancy in terms. *Apoptosis, pro-apoptotic*, and *programmed cell death *are highly-related, and all were picked as *i*Terms for gene *BAX*. We would like to explore methods to at least group them together. Additionally, eGIFT currently lists all sentences mentioning an *i*Term, without paying special attention to their redundancy. In the future, we would like to identify redundancy among sentences for a gene, and group together sentences with similar information.

### *i*Terms vs. controlled vocabularies in KBs

We now discuss four differences between *i*Terms and controlled vocabulary terms used to annotate a gene in knowledge bases.

#### Restricted terms (RT)

Controlled vocabularies are by definition restricted sets of terms and *i*Terms can be any combination of words found in the literature.

#### Restricted categories (RC)

Knowledge bases employ a set of controlled vocabularies corresponding to different categories (e.g. Gene Ontology for biological processes, molecular functions, and cellular components). But *i*Terms associated with a gene are not restricted to certain categories, although we classified *i*Terms into categories for organizational purposes.

#### Incomplete annotations (IA)

The association between a controlled vocabulary term and a gene is performed manually by curators. This is a time consuming task that is difficult to keep up to date and which leads to incomplete annotations. However, if mentioned frequently in the literature for a gene, a missed controlled vocabulary term could be picked as an *i*Term.

#### Association guidelines (AG)

Controlled vocabularies are not only intended to provide a set of terms with commonly agreed upon meaning, but also their association to genes should be agreed upon. For example, Gene Ontology and UniProtKB explain the situations in which an association can be made between a gene and a keyword or a GO term [31,32]. Because there is no predefined relation between *i*Terms and genes, we associate with an *i*Term all these sentences containing the *i*Term. This allows the user to determine the exact relationship between the *i*Term and the gene. The same reason also underlies our decision to name eGIFT's terms differently from keywords. As used in UniProtKB, "KeyWord lines provide information that can be used to generate indexes of the sequence entries based on functional, structural, or other categories" [32].

These four distinctions apply equally well to other systems like eGIFT that identify important terms based on their frequencies in the literature [3-5,10-12,14]. In fact, the last two distinctions also apply to systems that restrict their important terms to the ones found in controlled vocabularies [6-9].

By examining the terms found relevant during eGIFT's evaluation, we can see how the distinctions between *i*Terms and controlled vocabulary terms used by knowledge bases are realized.

Of the 694 relevant *i*Terms for the 40 genes used in eGIFT's evaluation, we found 235 (33.86%) terms associated with their corresponding genes in either one of the two knowledge bases we looked at (EntrezGene and UniProtKB). Of these, 180 (25.94%) *i*Terms were exact matches to some GO term, domain, pathway, phenotype, disease, ligand, or some other controlled vocabulary term used in these knowledge bases. The rest of 55 (7.92%) *i*Terms were partial matches. Note that *i*Terms are mostly unigrams and bigrams, while controlled vocabulary terms could be longer. Thus, the partial matches correspond to *i*Terms that were subsequences of a longer controlled vocabulary term annotated for the corresponding genes. For example, *i*Term *programmed cell death (pcd) *was partially matched to GO term *induction of retinal programmed cell death* for gene *BAX*. Sentences for this *i*Term in eGIFT discuss pcd of retinal cells.

This means that for nearly two thirds (or 459 of 694) relevant *i*Terms, we could not find a match with equivalent controlled vocabulary terms associated with these genes in the two knowledge bases. Since these *i*Terms were annotated as relevant by the evaluators, we attribute the remaining cases to one of the four distinctions between *i*Terms and controlled vocabulary terms in knowledge bases. Furthermore, 242 (34.87%) *i*Terms were included in the manually generated and curated EntrezGene summaries and GeneRIF sentences, or in UniProtKB's general annotations. Thus, while these terms appear in parts of the knowledge bases, summarizing the properties of the gene, they do not show up in the annotations based on controlled vocabularies.

We found *i*Terms exemplifying all forms of cases discussed above. In the case of association guidelines (AG), we point to *i*Term *phosphorylation *for gene BAD. This protein is phosphorylated, but does not initiate the process of phosphorylation. Thus, GO term *phosphorylation *cannot be associated with *BAD*. However, the phosphorylation state of *BAD *determines its role as apoptotic or anti-apoptotic and this is a crucial information, which is also noted in the summaries for gene *BAD*. In the case of incomplete annotations (IA), we note *i*Term *oligomerization *for gene *APAF1*. Sentences in eGIFT appear to suggest that this is an appropriate term to annotate for *APAF1*. An example of restricted categories (RC) is *osteoclasts *for gene *SPP1*, which was missed because currently knowledge bases do not associate cells with genes. And finally, *i*Terms *cellular permeability *for gene *OCLN *and *ww3 domain *for gene *NEDD4 *were not part of the annotations for these genes due to a restricted terms (RT) case.

A total of 217 (31.27%) relevant *i*Terms could not be found anywhere in the knowledge base entries of the 40 genes. Of 217 *i*Terms, 77 are in fact part of some controlled vocabulary used by these knowledge bases but not annotated for these genes. This could happen for two reasons: first, because the annotations for the genes are incomplete (IA), and second, because these terms do not follow the standard guidelines of associating them to the genes (AG). An example here of a missed annotation is GO term *cellular thermotolerance *for gene *CLPB *("Clpb is a highly conserved heat shock protein that is essential for thermotolerance in bacteria and eukaryotes" - PMID 11092876). Similar examples are *ion transport *for *ADD1 *and *diapedesis *for *F11R*.

The remaining 140 *i*Terms are terms that are not part of any controlled vocabulary. For this reason, these omissions must be due to restricted categories (RC) and restricted terms (RT) cases. For example, *i*Term *mitochondrial pathway *for gene *APAF1 *does not appear anywhere in its knowledge base gene entries, possibly because this term is not a controlled vocabulary term (RT) or a part of it. However, reading sentences containing this term in eGIFT, we learn that *APAF1 *"is an essential factor in intrinsic mitochondrial pathway of apoptosis activation" (PMID 19236791). Similar examples are *eh1 domain *for gene *GRO *(RT), *NA handling *for *ADD1 *(RC), and *happloinsuficiency *for *COL5A1 *(RC).

## Conclusions

We have developed a system, called eGIFT, that aims to assist life scientists in rapidly finding gene-related information from Medline abstracts. A user can consult a list of *i*Terms extracted by eGIFT and examine the sentences associated with these *i*Terms to quickly identify important concepts and how they are related to the gene. Moreover, *i*Terms are divided into different categories to allow users to hone in quickly to the type of information they seek.

eGIFT includes various components that are not found in many of the systems that mine important terms for genes. These include: (1) an eGRAB module, which gathers Medline abstracts that mention the given gene's names or synonyms and automatically filters out abstracts that are irrelevant to the gene; (2) a module that identifies the About Set for a gene by filtering those abstracts that appear to only mention the gene in passing; (3) mining of multi-word terms, including methods to avoid redundancy and problems with low frequency; (4) grouping words from the same lexeme family and using the frequency count of the lexeme in computing the score, rather than treating words as individual terms and computing their scores individually.

In this work, we conducted two experiments which suggest that eGIFT provides an accurate and comprehensive overview of a gene. The *recall *evaluation shows that eGIFT can assist in gene annotation, as it was able to identify 103 of 110 keywords used in the experiment. The *precision *study shows that on an average 17.5 out of top 20 *i*Terms returned for a gene are salient. In addition, we also evaluated two components of eGIFT: the eGRAB module, for which the recall and precision were high; and the selection of the About Set, which filters out with high accuracy the abstracts that are not focused on the given gene.

These evaluations also suggest three applications of eGIFT, which we intend to explore further:

First, although eGIFT's *i*Terms are not limited to controlled vocabulary terms, we believe eGIFT can be used to help make gene annotations more comprehensive. For several genes, using *i*Terms and their sentences, we manually found and confirmed with a GO term annotator that there might be more keyword annotations possible.

Second, eGIFT can be used to provide literature evidence tags, even for these GO terms that already exist in the gene's annotations. For instance, we noticed that 93 GO terms associated with the 103 keywords had an IEA evidence (Inferred by Electronic Annotation - no curator has checked the specific annotation to verify its accuracy). The sentences that eGIFT associates with the corresponding *i*Terms, in many cases, could lead to literature evidence used to change the IEA tags to more useful literature-based tags. We showed several such sentence/*i*Term pairs to a professional GO term annotator who agreed that these sentences were clear and likely indicators for upgrading the tags.

Finally, in looking at *i*Terms and their sentences, we often find sentences that precisely capture the relation between the *i*Term and the gene. We plan on finding a method to automatically identify such highly-informative sentences for the *i*Terms. The top *i*Terms and their highly-ranked sentences could be used to generate application/user-oriented short summaries.

## Availability and Requirements

eGIFT can be accessed freely at the following URL: http://biotm.cis.udel.edu/eGIFT. Users of eGIFT can browse the database, which currently includes 5,000 genes, or search for the gene of interest using a gene name or EntrezGene identifier. *i*Terms and sentences are pre-processed and updated monthly and new genes are added daily to the database.

## Authors' contributions

COT designed and implemented the text mining application and drafted the manuscript. CJS provided his expertise in molecular biology, participated in discussions, and helped to revise the manuscript. KVS conceived the overall study, participated in the design and coordination, and helped to draft the manuscript. All authors read and approved the final manuscript.

## Supplementary Material

Additional file 1 **Screenshot of eGIFT's *i*Terms for gene Groucho**1. This is an image of gene *Groucho*'s *i*Terms, as seen by accessing its webpage in eGIFT. Click here for file

Additional file 2 **List of genes used in the evaluation**. This file, provided in Excel format, includes a list of genes used in the evaluation of eGIFT. Provided inside are: EntrezGene ID, UniProtKB ID, short name of the gene, long name of the gene, the number of species in which this gene was found, the total numbers of a PubMed search for this gene, the Full Set (as identified by eGRAB), and the About Set. Click here for file

Additional file 3 **Results for eGIFT and eGRAB evaluation**. This file, provided in Excel format, includes the results of eGIFT's and eGRAB's evaluation for the 40 genes used in the experiments. Provided inside are: the short name of the gene, the recall/accuracy/precision of eGRAB, and the precision/recall of eGIFT's iTerms. Click here for file
