# Supplementary figures and images for: eGIFT: Mining Gene Information from the Literature
Source: BMC Bioinformatics. 2010 Aug 9;11:418. doi: 10.1186/1471-2105-11-418 (PMC2929241; doi:10.1186/1471-2105-11-418)

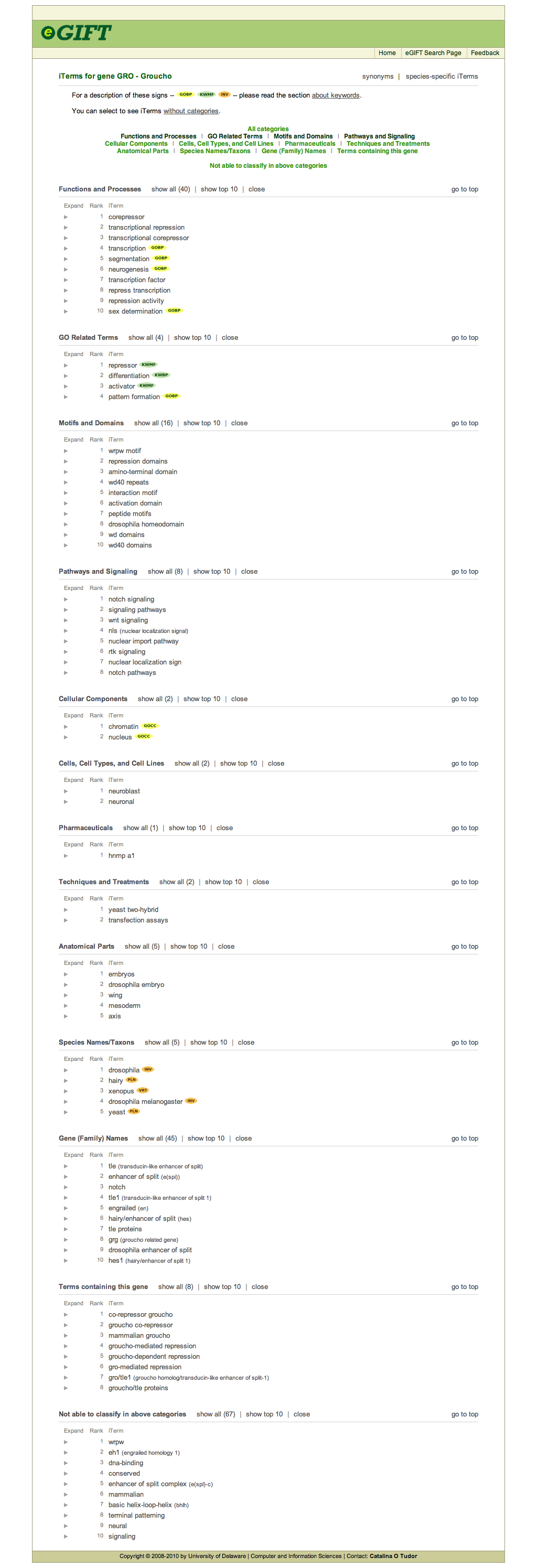

Supplement: Additional file 1 — Screenshot of eGIFT's iTerms for gene Groucho1. This is an image of gene Groucho's iTerms, as seen by accessing its webpage in eGIFT. [file 1471-2105-11-418-S1.PNG]
